# A Six-Step Model for Evaluation of Community-based Physical Activity Programs

**Published:** 2005-12-15

**Authors:** Levin Martin Sarah, Gregory W Heath

**Affiliations:** Division of Nutrition and Physical Activity, Physical Activity and Health Branch, National Center for Chronic Disease Prevention and Health Promotion, Centers for Disease Control and Prevention; Division of Nutrition and Physical Activity, Physical Activity and Health Branch, National Center for Chronic Disease Prevention and Health Promotion, Centers for Disease Control and Prevention, Atlanta, Ga; Dr Heath is now with the Department of Health and Human Performance, University of Tennessee at Chattanooga, Chattanooga, Tenn

## Introduction

Physical activity is a leading health indicator (1) and has numerous benefits, including reduced risk of coronary heart disease, hypertension, colon cancer, and diabetes (2). Regular physical activity can help prevent the onset of diabetes (3), even among those at high risk (i.e., those with impaired glucose tolerance [4]), and is part of diabetes self-management among people with the disease (5).

The Diabetes Prevention Program, a study funded by the National Institute of Diabetes & Digestive & Kidney Diseases, found that participants with impaired glucose tolerance who were assigned to an intensive lifestyle intervention reduced their risk of getting type 2 diabetes by 58%. On average, this group maintained their physical activity at 30 minutes per day, usually with walking or other moderate-intensity exercise, and lost 5% to 7% of their body weight (4).

It is likely that health educators at local health departments addressing diabetes and other chronic diseases will be asked to evaluate a physical activity program, because evaluation has a central role not only in improving programs but also in satisfying accountability requirements. A carefully planned evaluation can engage community members and build community capacity, and the results can be used to influence policy makers, share what works and what doesn't work with other communities, and help ensure funding and sustainability.

The Centers for Disease Control and Prevention (CDC) has published the *Framework for Program Evaluation in Public Health* (6), which recommends six steps for effective program evaluation: 1) engaging stakeholders, 2) describing the program, 3) focusing the evaluation design, 4) gathering credible evidence, 5) justifying conclusions, and 6) ensuring use and sharing lessons learned. In this article, we describe these six steps using a hypothetical example of physical activity programming aimed at diabetes prevention. For this example, we assume that the reader is a community-based health educator at a local health department.

## Selecting an Intervention

Before planning the evaluation, you should be familiar with strategies or interventions proven to increase physical activity at the population level. *The Guide to Community Preventive Services* (*Community Guide*) (7), available from www.thecommunityguide.org/pa/default.htm, includes eight recommended strategies that fall within three domains: informational approaches to increasing physical activity, behavioral and social approaches to increasing physical activity, and environmental and policy changes to increasing physical activity. Becoming familiar with these strategies is important background work. Because you may not have all the resources needed to carry out a population-based physical activity intervention, it is essential to work with partners. In fact, your role may be to influence others to carry out the program. One possible way to select the intervention strategy is by using the RE-AIM framework (information available from www.re-aim.org/[8]), which considers the *reach*, *efficacy*, *adoption*, *implementation*, and *maintenance* of public health interventions.

For this article, we selected the strategy of creating or enhancing access to places for physical activity, combined with informational outreach activities, from the *Community Guide*. (A description of this strategy is available from www.thecommunityguide.org/pa/pa-int-create-access.pdf.) This strategy involves the efforts of worksites, coalitions, agencies, and communities to change the local environment to create or improve access to opportunities for physical activity.

## Steps in an Evaluation of a Physical Activity Intervention

The CDC's six-step *Framework for Program Evaluation in Public Health* will be used to guide this step-by-step example (6). These six steps have been adapted for use in physical activity programs and published in the *Physical Activity Evaluation Handbook* (9), available from www.cdc.gov/nccdphp/dnpa/physical/handbook/index.htm. Because an evaluation is not worth doing if the information gleaned will not be used, utility is perhaps the most important standard for program evaluation. The other standards are feasibility (you cannot evaluate with resources you do not have), accuracy (you cannot evaluate with poor or invalid measures), and propriety (you cannot evaluate if you are not fair and ethical to everyone involved).

### Step 1: engage stakeholders

Important stakeholders for you as a health educator at a local health department are *partners*; these partners will carry out the intervention strategy. For an intervention to create or enhance places for physical activity, potential partners might include a city park, a shopping mall, the YMCA, the tourism bureau, and the community college. Also of great importance in terms of meeting the utility standard are *decision makers* — individuals who can use evaluation results to allocate future funds or cut programs. Examples might include the city mayor, the president of the community college, and the county-level director of parks and recreation.

You should invite all of the partner and decision-maker stakeholders to a meeting to describe the recommended strategy. The group should then discuss its role in making this strategy into a reality in its community and what evaluation resources it can offer.

Another group of stakeholders is the *participants*, individuals at high risk of developing diabetes. One way to engage participants is to invite them to a focus group or town meeting. At such a gathering, their ideas about the program can be assessed and used to refine the program to meet their needs. For the "creating access" strategy, for example, the participants can reveal what physical activity offerings would interest them and what venues might best reach them with information about these offerings.

### Step 2: describe the program

The partners should be invited back for a second meeting to work on a logic model to depict graphically the proposed relationship between activities and expected outcomes. In this step, the work pertains to planning both the intervention and its evaluation. On the basis of step 1, the health educator is able to share the list of activities discussed at the first meeting and indicate which ones seem more popular or less popular among participants in the focus groups or town meetings.

To begin creating a logic model, the partners can divide the activities into two columns, early activities and later activities. Then the group should discuss outcomes they can realistically expect from the proposed activities. One outcome that seems obvious is an increased level of physical activity of residents, but there are more immediate and targeted outcomes that may precede such a behavioral change, such as increased opportunities for physical activity, increased awareness of physical activity offerings, and limiting of the target population to those at risk for developing diabetes, diabetes complications, or both. For diabetes care there may be outcomes even later than increased levels of physical activity, such as decreased levels of hemoglobin A1c (HbA1c) and, eventually, decreased incidence of diabetes morbidity and mortality. It is the role of the health educator to insist that the group set short-term objectives that include measurable outcomes. Objectives should be SMART — that is, *specific*, *measurable*, *achievable*, *relevant*, and *time-bound*. (See Appendix 4 in the *Physical Activity Evaluation Handbook* [9].) After the activities and the outcomes have been placed in sequence, the logic model begins to take shape. Inputs (i.e., resources to carry out the activities) can be added to the far left, and an overarching goal can be added to follow from long-term outcomes, as shown in the Figure.

**Figure:**
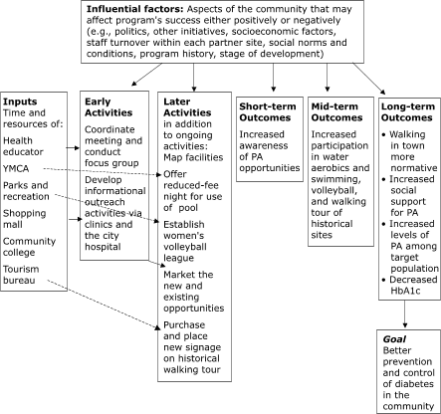
Example of a logic model for an intervention to create or enhance access to physical activity (PA) combined with informational outreach activities.

**Table:** 

### Step 3: focus on evaluation

As a health educator, you want to be sure that the evaluation is useful not only to your health department but also to the partner organizations that help to implement the program. To ensure buy-in and later use of the evaluation, ask stakeholders to develop questions that they would like to have answered. For example, the YMCA staff may want to know whether their membership increases. Increased membership becomes an outcome in the logic model. Note that the logic model can be made from right to left, that is, by asking, "How will we increase membership?" and then proposing some activities that might lead toward that outcome. Or it can be made from left to right, by asking, "Why are we doing that?" and then stating the expected outcomes of such activities. Either way, a focused evaluation will be one that poses questions based on the program and one that results in answers that serve the purpose of the evaluation. The purpose often will be to improve the program; other purposes may include gaining insight and assessing program effects. Defining your purpose is an important component of this step.

In our example, the stakeholders have already agreed on a logic model in step 2 (Figure), and so they can use it to focus their questions. They might decide to ask both process and outcome evaluation questions. Process questions relate to the inputs and activities, and outcome questions relate to the expected outcomes. It is possible to generate a long list of possible questions from the logic model, but then the list needs to be prioritized. Evaluating all questions may not be essential or even feasible. The stakeholders should remember the purpose of the evaluation and decide what would be useful for decision makers in prioritizing the list of questions. Examples of process and outcome questions include the following:

**Process**

- Are the proposed activities being carried out by the partners? If not, why not?
- What seem to be the most popular activities, and why?
- Are we reaching individuals at risk for diabetes?

**Outcome**

- Did awareness of opportunities increase?
- Did participation increase? Why or why not?
- Did HbA1c levels decrease among the population with diabetes?

### Step 4: gather credible evidence

To answer the questions posed in step 3, evidence needs to be collected. How much evidence (quantity) and what kind of evidence (quality) are central to feasibility and accuracy. There must be a balance between collecting enough data and assuring it is of high quality. Sometimes a mix of quantitative and qualitative data will help achieve that balance: quantitative data can provide the numbers you need to answer some questions (e.g., participation rates), and qualitative data can help you understand why you got those numbers (e.g., interview a few who participate and a few who do not to learn why). Data are available from people, documents, observations, and existing information.

The Table provides a guide to collecting data for process and outcome questions by indicators, data sources, and performance measures. Indicators are what answer the question, data sources are the methods by which you collect data about the indicators, and performance measures are the outcomes you would like to achieve. It is helpful to have more than one indicator and more than one data source to answer each evaluation question. Using multiple indicators and data sources is often called *triangulation* and is recommended to increase accuracy. There are many tools available for collecting physical activity data. (See Appendix 5 in the *Physical Activity Evaluation Handbook* [9].)

### Step 5: justify conclusions

There are three parts to this step: 1) analyze the data, 2) interpret the results, and 3) make judgments about the program. Having the performance measures helps to justify your conclusions. Perhaps a community college student needs an internship. You can hire him or her (often without financial compensation) to help with the evaluation. With guidance from you and the supervising professor, the student can analyze the data. Analysis for some questions will be easier than for others. For example, the difference between participation rates preintervention and postintervention is simple math, whereas analyzing focus group and interview data takes more time because all of the text must be read and common themes identified to answer the appropriate evaluation question.

After the analyses, you should convene a meeting of stakeholders to go over the results. Talk about possible alternative explanations to the findings of the evaluation. Discuss the limitations. One common limitation is having no control community; if possible, use a selected community as a basis of comparison in a quasi-experimental design. (For more on experimental designs, see the *Physical Activity Evaluation Handbook*, p. 26 [9].) Compare the results with the performance measures, and make judgments based primarily on that comparison. If you realize you did not achieve a performance measure, decide if you are willing to say that the program failed. It could be that almost every indicator showed improvement. With the decision makers present, the group can decide which results matter most and use those to summarize their findings to share with the community at large.

### Step 6: ensure use and share lessons learned

The findings can be printed in the local newspaper, which, in our case, has the beneficial effect of increasing awareness of physical activity opportunities even further. The best features of the program should be highlighted. You can send the findings electronically to the CDC, where they can be widely distributed through the Physical Activity Listserv; in addition, examples of state physical activity programs can be posted on the State-based Physical Activity Directory at http://apps.nccd.cdc.gov/DNPAProg/.

To ensure use of your evaluation findings, formulate action-oriented recommendations. To help share lessons learned, consider your audience: use appropriate communications strategies and consider the most effective format for information (e.g., report, fact sheet, oral presentation) and venue (e.g., Web site, television, news media, town hall meeting).

## Conclusion

Although the principles of evaluation transcend topical areas, we hope that this step-by-step guide provides insight and examples for evaluating physical activity programs. There are evidence-based strategies for promoting physical activity in a community setting, and there are diabetes programs across the nation that could implement these strategies by engaging partners and initiating systems change. In our examples for creating access to places for physical activity, systems were engaged, and the strategies depended on these system changes. Partner organizations benefit by learning principles of evaluation that they can use for continuous quality improvement. Program evaluation plays a key role in ensuring success and sustainability of these programs. 

## Figures and Tables

**Table:** Guide to Data Collection for Sample Intervention to Create or Enhance Access to Physical Activity, Combined With Informational Outreach Activities

**Evaluation Question**	**Indicators**	**Data Sources**	**Performance Measure**

**Process**			

Are the proposed activities being carried out? If not, why not?	Presence of classes Activity leaders' impressions	Observation Interview	100% of proposed activities happening
What seem to be the most popular activities? Why?	Participation rates Opinions of target population	Sign-in sheets^a^ Focus group of potential participants	20 per swim session, 12 per volleyball game or practice, 10 visitors per week for historical walk map, etc.
Are we reaching those at risk for diabetes complications?	Names of participants	Sign-in sheets crossed with medical records	75% of participants for enhanced activities will be from the target population

**Outcome**			

Did awareness of opportunities increase?	Percentage aware of opportunities	Survey of all persons at risk for and with diabetes seen in the clinics and the city hospital	80% awareness among target population by 6 months
Did participation increase? Why or why not?	Number of participants	Preinformational outreach participation rates versus postoutreach rates	50% increase in participation rates per site (YMCA, tourism bureau, community college)
Did hemoglobin A1c (HbA1c) levels decrease among population with diabetes?	Results of finger stick	Blood test in HbA1c analyzer^a^	10% reduction in mean HbA1c level among participants with diabetes within 1 year

a Informed consent obtained for participation through recruitment at local clinics and hospital.
